# Visual metaphor of sadness in poetry comics: a socio-cognitive perspective

**DOI:** 10.3389/fpsyg.2023.1264068

**Published:** 2023-10-30

**Authors:** Suijun Wen, Zenan Zhong, Shukun Chen

**Affiliations:** School of Foreign Languages and Cultures, Guangdong University of Finance, Guangzhou, China

**Keywords:** poetry comics, visual metaphor, sadness, socio-cognitive perspective, Chinese cultural context

## Abstract

Earlier literature on conceptual metaphor studies has extensively examined verbal metaphors of sadness in different text types and with cultural variations. However, there has been by far limited research on the visual metaphor of sadness. Adopting a socio-cognitive perspective, this study investigates the conceptual metaphor of sadness in the exemplary case of Chinese poetry comics drawn by Cai Zhizhong. The findings reveal that (1) BEING SAD IS BEING CONFRONTED WITH NATURAL FORCE and BEING SAD IS BEING PHYSICALLY ISOLATED are the two most frequently occurring visual metaphors across the panels; (2) all the visual metaphors at play can be explained according to the conceptual metaphor theory; (3) SADNESS IS BITTERSWEET FOOD OR DRINK and BEING SAD IS BEING PHYSICALLY ISOLATED are two additional kinds of sadness metaphors identified; and (4) the visual metaphors of sadness with Chinese cultural variations are rooted in mainstream Chinese cultural philosophies in the relevant period of history. The article also discusses the underlying mechanisms of the investigated visual metaphors in the Chinese culture by unveiling three cultural characteristics in the particular context.

## 1. Introduction

Sadness is an emotion “generally associated with the appraisal of permanent loss.” (Bonanno et al., 2008, p. 798). This type of emotion is found widely expressed in classical Chinese poetry, especially in *Song Ci*. *Song Ci*, as one of the treasures of ancient Chinese literature, is a linguistic form where the Chinese expressively incorporate emotions, such as sadness, “of great depth and intensity [.] publicly in poetry” (Whincup, 1987) and where sadness is always represented by figurative expressions (Feng and Lin, 2017). Such figurative expressions used to denote various aspects of emotion concepts could be metaphorical expressions, which according to Lakoff and Johnson (1980) are “the manifestation of conceptual metaphors.” Through conceptual metaphor manifestation, abstract and complex emotions are transformed into direct and concrete experiences. As far as the metaphorical expression of sadness in Chinese poetry is concerned, the types of conceptual metaphors could be “SADNESS IS LIQUID,” “SADNESS IS PHYSICAL OBJECT,” and “SADNESS IS PLANT” (Feng and Lin, 2017).

To the essence of conceptual metaphor, Lakoff and Johnson (1980, p. 153) theorize that “[m]etaphor is primarily a matter of thought and action and only derivatively a matter of language.” In other words, in addition to language, other communicative semiotic devices such as images, colors, and spatial layouts can also be used to express metaphorical concepts (Forceville, 2005). The viewpoint has been demonstrated by the pioneering work of Forceville (2006) study on the visual metaphors of the emotion of anger (c.f. Zhong et al., 2023).

In this sense, how the emotion of sadness could be metaphorically visualized is an issue worth exploring. The Chinese *Song Ci* being discussed was produced in the Song Dynasty, which went through political turmoil, wars, and eventual demise. Such circumstances gave rise to the themes of *Song Ci* mainly reflecting poets’ discouragement toward reality and the future, as well as disappointed misanthropy (Wang, 2000), fostering a typical type of Chinese poetry composed with melancholic themes. Such poetry, in recent years, has been compiled into a comic book by a famous cartoonist, Cai Zhizhong (hereafter Cai) or Tsai Chih-chung. He is devoted to visualizing Chinese classic sinology, producing works that have been translated into over 20 languages and published in more than 49 countries worldwide, with sales reaching over 40 million copies (Bi and Zhu, 2011, cited in Zhong et al., 2021). Regarding *Song Ci*, his work endeavors to portray the emotion of sadness through comic images. The earlier study of one of Cai’s works shows that the metaphors in poems describing things and circumstances that the poet perceives are transformed metaphorically into comics (Chen and Zhong, 2022). The present study is thus an attempt to examine what types of conceptual metaphors the images employ to express the emotion of sadness.

Our study works under the conceptual metaphor theory (hereafter CMT). It brings “two distant domains (or concepts) into correspondence with each other” (Kövecses, 2000, p. 4). Conceptual metaphors of emotions have been investigated in wide-ranging text types, including both verbal and visual discourse, such as advertisements, news, and financial reports (e.g., Kövecses, 1986, 1990, 2000; Yu, 2009; Abbott and Forceville, 2011; Khajeh et al., 2013; Nguyen, 2013; Türker, 2013; Patowari, 2015; Luo, 2016; Csillag, 2017). Those investigations have identified how abstract feelings are mapped from the source domain of physical entities and have implied further research issues, such as cultural factors in constructing metaphors. Therefore, more research is needed to examine the metaphors of emotions in other contexts, for example, visual metaphors of sadness in the Chinese context of the present study, to provide a more comprehensive understanding of the visual metaphors of emotions. Our investigation of Cai’s works, hence, would contribute to the line of research on visual metaphors of sadness. Following this line of research, this study adopts a socio-cognitive perspective focusing particularly on the visual representation of conceptual metaphors of sadness, in an attempt to answer the following three questions:

1. What source domains of conceptual metaphors can we recognize in the visual representation of sadness based on the comic discourse from *Song Ci*?
2. How can we explain these source domains from the perspective of conceptual metaphors?
3. How are the visual metaphors of sadness represented in the Chinese cultural context in a graphic narrative?

## 2. CMT on visual metaphor of emotion

The study of emotion in CMT has been flourishing along the way. This particular line of research recognizes the origin of emotion language in physical responses, either mental or physical (see, e.g., LeDoux, 1996; Glenberg et al., 2005). It follows that various conscious feelings share similar and for some the same source domains (Kövecses, 2000), which has been evidenced by scholarship on a wide range of text types. For instance, Díaz-Vera (2014) investigates the set of emotion-related personal names in the Domesday Book and shows not only its wide presence but also the patterns related to gender distribution and lexical combinations. Hansson and Norberg (2012) studies Ellen Wood’s sensation novel *East Lynne* (1861–1862) and analyzes the part of emotion metaphors in constructing gender roles, which is similar to the path followed by Abbott and Forceville (2011) though the latter focus on the effect of visual emotion metaphor. In addition, metaphors of emotion function with specific cultural elements unique in different cultural contexts (Kövecses, 2005, p. 235). Khajeh et al. (2013), for example, extend the conceptual metaphor of SADNESS IS FLUID IN A CONTAINER and propose that sadness can also be an emotional experience that involves heat in Persian, thereby adding an extensive cultural characteristic that SADNESS IS A HOT FLUID IN A CONTAINER by drawing evidence from their cultural notions and models.

The verbal metaphor of emotion has been shifted to be presented in the same way in visual modes, though often with modifications or extensions (Kövecses, 2015). For example, ANGER IS THE HEAT OF A FLUID IN A CONTAINER is a dominant metaphor identified by Kövecses (1986, 2000), but Forceville (2005) has worked out a number of different ways, such as smoke, bulging eyes, and red/pink face to represent anger metaphorically in pictorial signals in La Zizanie (1970). The conceptual metaphor of anger in the manga is further investigated by Abbott and Forceville (2011), adding another major source domain of “loss of hands.” However, there has been by far insufficient account of the conceptual metaphor of sadness in visual discourse in a Chinese context. As we are identifying the representative images of sadness in Cai’s comics in our pilot study, it turns out that the conceptual metaphors there have been displayed in a similar way to findings in earlier literature, with expected cross-cultural variations (Kövecses, 2000). Conceptual metaphors function in a way where emotions are mapped from source domains to target domains. However, the world of comics has been constructed with not only signs that point to the same meanings as the metaphors listed but also cultural variations in the visual metaphors. In Shinohara and Matsunaka (2009), it is argued that emotion is an outer force rather than an inner force in Japan to understand the visual emotion metaphors in its culture. This is also true in the poetic world of Chinese culture where it is often expressed that human being, or rather the emotion of human being, is closely intertwined with the rhythms of nature (Tian, 2010, p. 65).

Therefore, this article is an attempt to contribute to the existing body of literature by not only examining the mechanisms of established source domains in the visual discourse but also adding insights into CMT in the Chinese cultural context. We will show how to look into the sadness metaphors in the poetic world with a visual approach, both quantitatively and qualitatively, and single out the reasons behind the identified metaphors by examining the theoretical and cultural mechanism that functions underneath the visual metaphors in question.

## 3. Theoretical framework and methodology

### 3.1. Theoretical framework

Conceptual metaphor theory declares metaphor is “understanding and experiencing one kind of thing [concept] in terms of another [concept]” (Lakoff and Johnson, 1980, p. 5). One of the concepts is more abstract and complex, and the other is more basic and concrete. The abstract concept is considered as the target domain and the basic concept is considered as the source domain. They are correlated by “a variety of human experience, including correlations in experience, various kinds of non-objective similarity, biological and cultural roots shared the two concepts” (Kovecses, 2010, p. 79). Thus, the nature of CMT is based on the “perceived similarities.” Another declaration of CMT is that metaphor is “primarily a matter of thought and action, and only derivatively a matter of language” (153), which implies metaphor is not verbal in nature. The primary declarations signify the metaphorical patterns spread through visual and multimodal discourse (Forceville, 2016).

This current study draws on Kövecses (2000) model of the conceptual metaphor of emotion. Emotion metaphors figure prominently as one of the important research domains within CMT. According to Kövecses (2000, p. 186), “[a]n emotion concept typically evokes content pertaining to all aspects of experience: social, cognitive, and physical.” The meaning “consists of two complementary parts: a basic image-schema of force and cultural content” (Kövecses, 2000, p. 189). The former, also called “force-schema,” is basically viewed as a universal “semantic primitive.” For example, being SAD universally corresponds to the orientation DOWN. The latter, however, is culture-specific, relating to the factors that constitute a culture. With some modifications of Barcelona (1986) and Kövecses (2000, p. 25–26) presents a set of typical types of source domains mapping sadness by language expressions, such as SAD IS DOWN, SAD IS DARK, SADNESS IS A NATURAL FORCE, and SADNESS IS A LIVING ORGANISM. Regarding visual metaphor, Kovecses (2010, p. 64) indicates cartoons are a rich source for metaphor mappings and are often portrayed in a “literal” way. In the comic image, a man being sad can be depicted as a drooping posture metonymically representing DOWN, which constructs an orientational metaphor, SAD IS DOWN. As mentioned above, the concepts of emotion also relate to socio-cultural factors. From Kövecses (2000, p. 190) perspective, “the conceptualization and experience of emotional feelings are structured by cultural models.” The perspective implies there would exist cultural variations in emotion metaphor patterns, in either language or visual discourse.

Therefore, investigating the patterns of visual metaphors of sadness in different cultural contexts is essential for enriching metaphor studies. Drawing on the key notions provided in CMT, our goal is to investigate how the adapted comic book of Chinese *Song Ci* maps the emotion of sadness. Specifically, the investigation is to answer the three questions provided in section “2. CMT on visual metaphor of emotion.” The following provides our methodology and data.

### 3.2. Data and methodology

**FIGURE 1:**
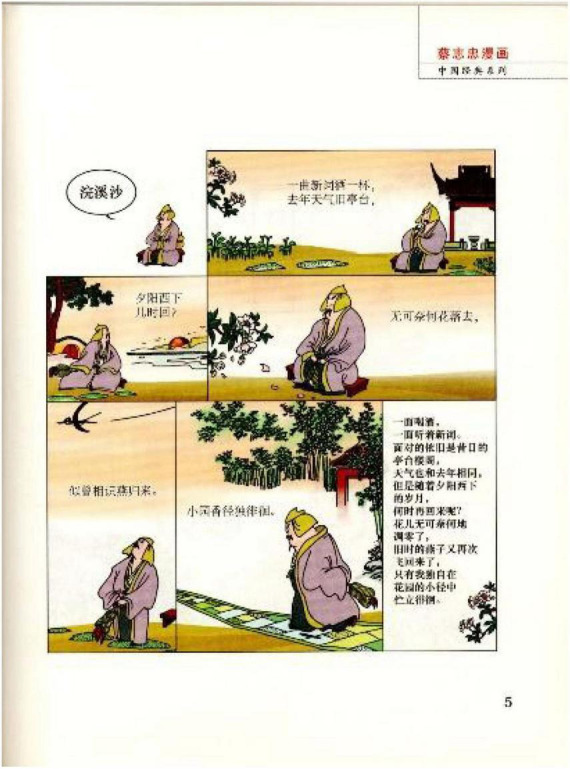
Example of a piece of Ci in Cai’s comics (Cai, 2014). Images reproduced with permission from Shan Dong People Press.

We have selected to study 225 panels in one of Cai’s comic books named *Song Ci: Hua Jian De Xi Su* (Song Poems, the Chitchat in the Flowers). The content in this book is unique in Cai’s series of Chinese classic series that focuses exclusively on the literary genre of *Song Ci*, which covers all the *Song Ci* comics drawn by Cai in the first 72 pages. As is similar to the other comic works named *Tang Shi San Bai Shou: Qian Gu De Jue Chang* (Three Hundred Tang Poems: Songs of Millenniums) by Cai (c.f. Chen and Zhong, 2022), each piece of Ci is turned into a comic strip, each made up of a verbal and a visual part, ended up with an explanatory note given by Cai (see Figure 1). As the length of Ci is a variable determined by Cipai, a sub-category of poetic style, the total number of panels in each comic strip varies, consisting of five, eight, or more panels.

To facilitate the understanding of readers, Cai also provides a simplified explanation in modern language about what is meant by the piece of poetry in the last panel. It is interesting to note that most of these comic works of *Song Ci* contain visual depictions of the emotion of sadness. Our criteria for including an image in our analysis from the pile of panels in the book were as follows:

1. The image could be understood as being about “sadness”—which transpired from the visual context, explanations by Cai, the comic artist, and/or observations in corresponding poetic lines.
2. The image was from all panels but the first and the last in each piece of poetry, which means they together form a graphic narrative, enabling the analysis of the narrative flow in some detail.

We first imported all the selected images into UAM Image Tool 2.1 (available at),^1^ an operational interface and conducted a quantitative investigation in the data set of images meeting our criteria and presented them under the established source domains including SADNESS IS DOWN, SADNESS IS DARK, BEING SAD IS BEING CONFRONTED BY A NATURAL FORCE, BEING SAD IS BEING CONFRONTED BY AN OPPONENT, and SADNESS IS LACK OF HEAT (Kövecses, 1986, 1990, 2000) and two added source domains of SADNESS IS BITTERSWEET FOOD OR DRINK and BEING SAD IS BEING PHYSICALLY ISOLATED which we identify as particular source domains that exist in a visual and Chinese cultural context.

**FIGURE 2:**
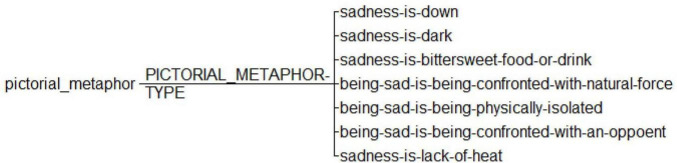
Annotation scheme for visual metaphors in selected images.

We have coded source domains in different images separately and in cases where one image contains more than one source domain. We also code the occurrence of each source domain therein as one. In addition to the quantitative discussion, we will discuss specific examples of visual metaphors under the framework of CMT, with a particular note on the adoption of Chinese cultural elements in visual representation. All the seven source domains adopted in our analysis are presented in Figure 2. This methodology has been proven applicable in the discussion of culture-specific cases of visual metaphors of emotion. For instance, Abbott and Forceville (2011) propose a new type of visual representation of emotion in manga, which is loss of control is loss of hands as substantiated by both qualitative and quantitative evidence in selected images with CMT. Similarly, using an idealized cognitive model, Eerden (2009) examines the visual metaphor of anger in the Asterix album La Zizanie in both static and moving images.

## 4. Quantitative findings

**FIGURE 3:**
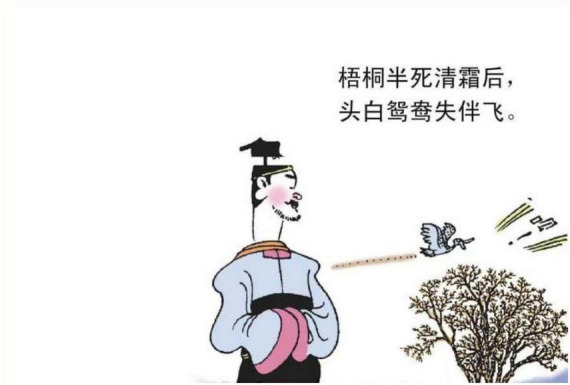
The second panel on Cai (2014, p. 29). Images reproduced with permission from Shan Dong People Press.

**FIGURE 4:**
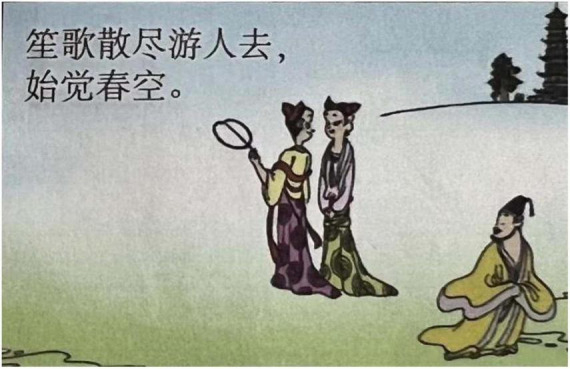
The third panel on Cai (2014, p. 9). Images reproduced with permission from Shan Dong People Press.

**FIGURE 5:**
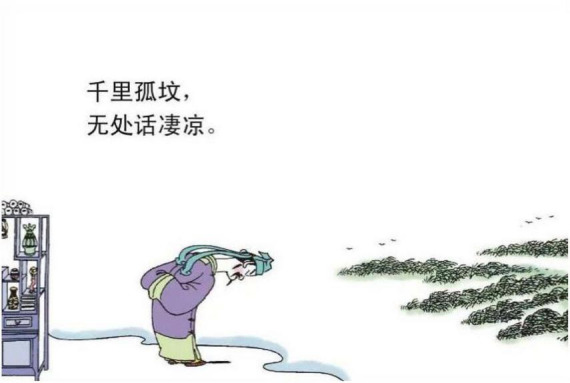
The second panel on Cai (2014, p. 11). Images reproduced with permission from Shan Dong People Press.

From the information given in Table 1, we can easily identify the two most frequent source domains in Cai’s work are BEING SAD IS BEING CONFRONTED WITH NATURAL FORCE (48) and BEING SAD IS BEING PHYSICALLY ISOLATED (44), each constituting more than one-third of the total number of domains of sadness with 34.29 and 31.43%. There are two possible reasons for this distribution. First, the Chinese follow a tradition of worshiping heaven whereby the presence of natural phenomena, such as wind, thunder, and storms, can be used to provoke the emotion of sadness. Second, as Chinese culture is known to boost in an environment where collectivism is widely valued (Hofstede, 2001, p. 250), the departure of any organism is always used to represent the emotion of sadness, such as the departure of birds and humans (often with small dots behind as in Figures 3, 4). The isolation by a division line or other substances is also a type of BEING SAD IS BEING PHYSICALLY ISOLATED, as in Figure 5. Here, we need to mention that Figure 5 also exists another type of metaphor: SADNESS IS DOWN. SADNESS IS DOWN, as a universally recognized domain, records a frequency of 39, and occupies a large portion of 27.86%. SADNESS IS BITTERSWEET FOOD OR DRINK is another source domain with cultural characteristics, while it only registers 3.57%, a much lower proportion than the first two ones. The shares of the remaining three source domains are minimal, with 1.43% for the domain of SADNESS IS DARK almost equaling the sum of the last two BEING SAD IS BEING CONFRONTED WITH AN OPPOENT and SADNESS IS LACK OF HEAT with 0.71% each.

**TABLE 1:** Distribution of source domains of sadness in all images.

Source domains	Frequency	Percentage
SADNESS IS DOWN	39	27.86%
SADNESS IS DARK	2	1.43%
SADNESS IS BITTERSWEET FOOD OR DRINK	5	3.57%
BEING SAD IS BEING CONFRONTED WITH NATURAL FORCE	48	34.29%
BEING SAD IS BEING PHYSICALLY ISOLATED	44	31.43%
BEING SAD IS BEING CONFRONTED WITH AN OPPONENT	1	0.71%
SADNESS IS LACK OF HEAT	1	0.71%
Total	140	100%

**FIGURE 6:**
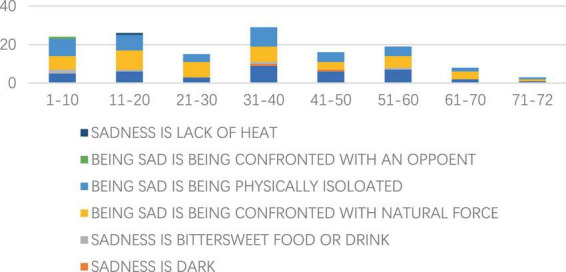
Distribution of source domains in every 10 pages.

According to Figure 6, BEING SAD IS BEING CONFRONTED WITH NATURAL FORCE amounts to be a most reoccurring source domain across all the pages, with consistent records of frequency in every 10 pages. While BEING SAD IS BEING PHYSICALLY ISOLATED features a total number that comes close as the second dominant domain, its presence throughout the pages is not as stable, with pages 21–30 in particular witnessing no such source domain at all. SADNESS IS DOWN, the third most identified, is also a consistent and frequent source domain on most pages.

The most evenly distributed source domain among the remaining four less frequent ones is SADNESS IS BITTERSWEET FOOD OR DRINK, which means wine, along with other alcoholic drinks are also popular devices to counter the unleashed sorrow in a Chinese context. BEING SAD IS BEING CONFRONTED WITH AN OPPOENT and SADNESS IS LACK OF HEAT are also possible concrete representations of sadness, though they are only adopted to express this emotion in the first 20 pages and pages 31–50.

In the following section, we will conduct further analysis to reveal the functioning of the source domains of sadness identified in the quantitative analysis, with particular notes on those taking up the largest percentages and bearing Chinese cultural variations.

## 5. Discussion

From the quantitative analysis above, we can see that the emotion of sadness is constructed by seven types of source domains in the comic book. Among these, BEING SAD IS BEING CONFRONTED WITH NATURAL FORCE, BEING SAD IS BEING PHYSICALLY ISOLATED, and SADNESS IS DOWN are the three most prominent metaphors. This section attempts to scrutinize how the source domains are deployed to be mapped onto the target domain of the emotion of sadness. We will talk about the domains in relation to the three categories of CMT, namely, orientational, ontological, and structural metaphor.

### 5.1. Being sad is being confronted with natural force

“BEING SAD IS BEING CONFRONTED WITH NATURAL FORCE” in the comic book is either an ontological metaphor or a structural metaphor.

The ontological metaphor involves living organisms and substances. The experience with the living organism and substance helps us to understand another concept, to “refer to them, categorize them, group them and quantify them” (Lakoff and Johnson, 1980, p. 25), and, in the present investigation, to view the emotion of sadness.

**FIGURE 7:**
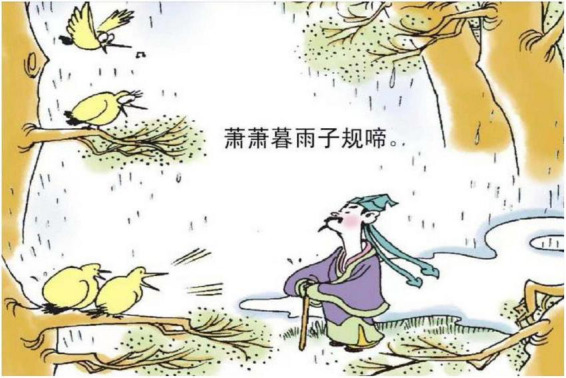
The second panel on Cai (2014, p. 18). Images reproduced with permission from Shan Dong People Press.

**FIGURE 8:**
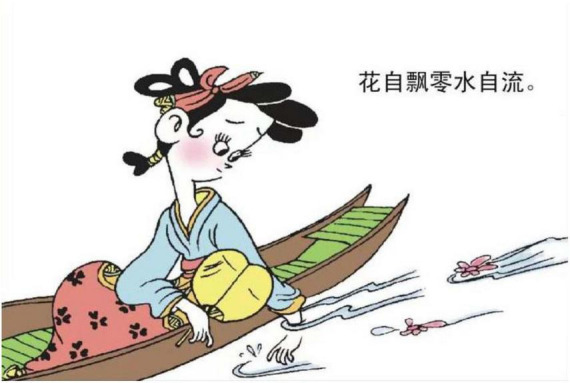
The first panel on Cai (2014, p. 39). Images reproduced with permission from Shan Dong People Press.

The living organism is a general term for living objects in nature. The living organism metaphor in the study includes birds, such as cuckoos, mandarin ducks, and plants. A Cuckoo in Figure 7 is a type of bird whose sound is perceived as mournful. The experience provides the basis for understanding the emotion of sadness, which is like hearing the mournful sound of the cuckoo. As to the Mandarin ducks in Figure 3, as they always appear in pairs, a single duck is viewed as being lonely, being miserable. Therefore, the experienced feature of a single mandarin duck could metaphorically structure the emotion of sadness. The plants that are employed to represent the emotion of sadness are the fading flowers, falling leaves, and pine trees. The flowers or the leaves that are depicted in a fading or falling state as in Figure 8 reflect being without energy. It supplies the ground to perceive the target domain of sadness.

**FIGURE 9:**
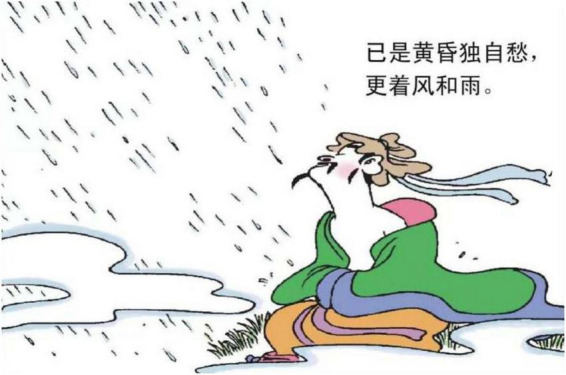
The second panel on Cai (2014, p. 54). Images reproduced with permission from Shan Dong People Press.

**FIGURE 10:**
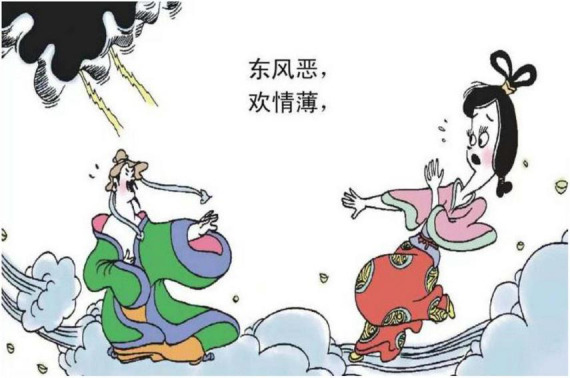
The second panel on Cai (2014, p. 52). Images reproduced with permission from Shan Dong People Press.

**FIGURE 11:**
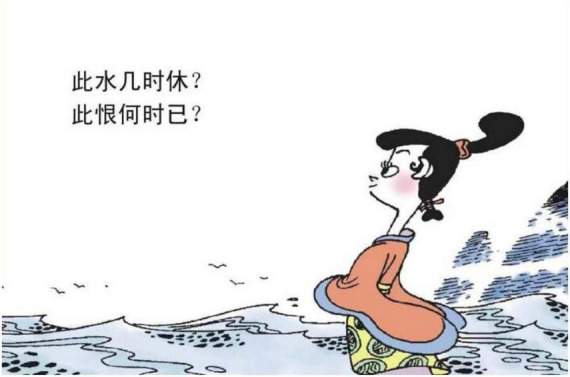
The third panel on Cai (2014, p. 70). Images reproduced with permission from Shan Dong People Press.

Substance metaphors could be classified into natural substances and material substances. In the images, many natural substances are employed to evoke the emotion of sadness, specifically, rain, frost, lightning, river, and moon. Take rain in Figure 9 for instance. The drizzle of rain is perceived as dense, like mist, like fog. The feature could be physically experienced, and it interrelates with the aspect of sadness emotion which is confusing. Lightening is another type of natural phenomenon as is shown in Figure 10, which is strong, forceful, and sharp. Based on the basis of this type of physical experience, the anxiousness of sadness could be provoked. The river or sea, which is long and wide, could sometimes have waves, mimicking the potentially fluctuating mood experienced in sadness emotion. In Figure 11, the experience of roaring waves could reflect the choppy state of sadness and the endlessness of the river activates the semantic mapping of the infinite sadness.

**FIGURE 12:**
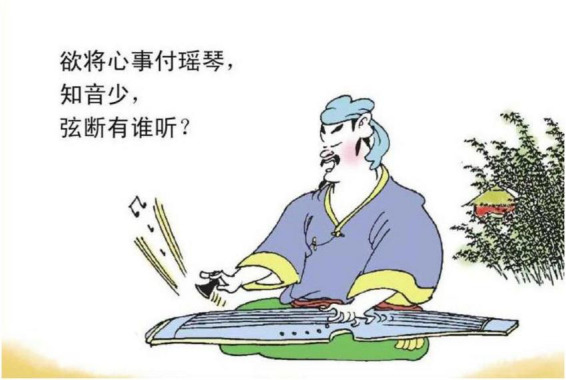
The fourth panel on Cai (2014, p. 49). Images reproduced with permission from Shan Dong People Press.

**FIGURE 13:**
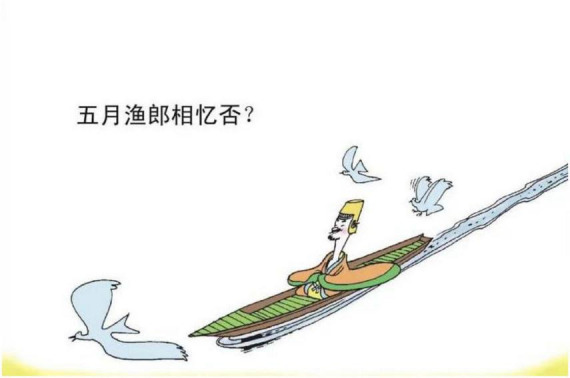
The second panel on Cai (2014, p. 32). Images reproduced with permission from Shan Dong People Press.

Material substances are also found, including the *Guqin* (plucked stringed instrument), tombstone, narrow boat, and red candle. *Guqin* is a traditional instrument in China with a deep tone, expressing a sense of sorrow, as shown in Figure 12. The feature is always considered to evoke the emotion of sadness, particularly in the wanderers in border areas. Therefore, the sound of the *Guqin* is frequently related to the understanding of sadness. Narrowboat also appears many times in the book, an example of which is Figure 13. The feature of being long in length and narrow in width contributes as the ground maps the suffocating feeling of sadness.

**FIGURE 14:**
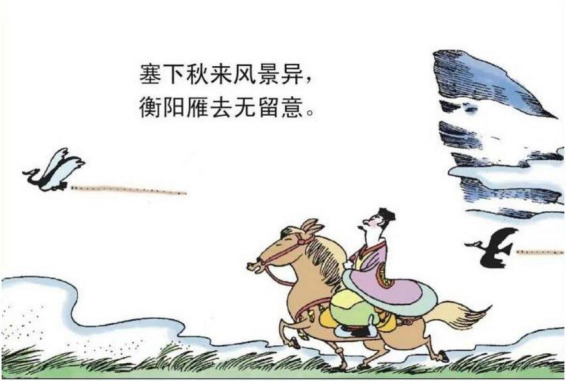
The first panel on Cai (2014, p. 2). Images reproduced with permission from Shan Dong People Press.

The structural metaphor of BEING SAD IS BEING CONFRONTED WITH NATURAL FORCE is the journey metaphor. A journey metaphor is an event structure metaphor that is based on a path schema, containing travelers, paths, ways, directions, and traveling companions. Factors during the journey provide the basic cognitive structure. For example, in Figure 14, the traveler is riding on a horse in the borderland. The image of the walking horse and flying wild geese activates the “path” scene in the journey metaphor, which represents the feature of desolation on the journey. Along the path, it creates mappings from the source domain of desolation on the journey to the target domain of sadness emotion.

### 5.2. Being sad is being physically isolated

**FIGURE 15:**
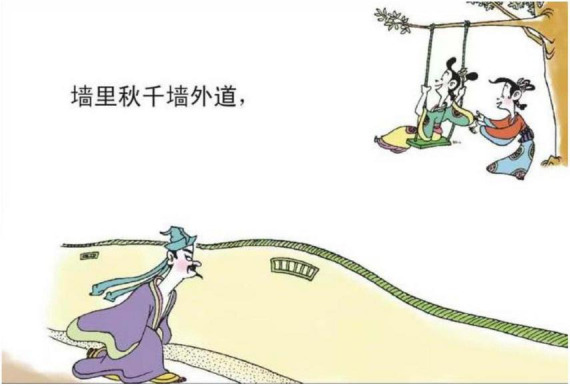
The fourth panel on Cai (2014, p. 19). Images reproduced with permission from Shan Dong People Press.

“BEING SAD IS BEING PHYSICALLY ISOLATED” is another major type of sadness metaphor, which regarding CMT categories, could be a horizontal orientational metaphor, related to the polar position of “in-out.” In Figure 15, there is a fence between a male protagonist and two female protagonists. The facial expressions of the two female protagonists metonymically represent happiness. The fence here isolates the male and the two female protagonists, constructing a container where there is inside and outside. In the Chinese cultural system, WE is highly valued, and IN is considered to be included in the group and safe, while OUT is considered unsafe, being excluded. The fence thus represents isolation metaphorically signifying the loneliness of sadness. Figure 4 is another example belonging to the case. It is also a horizontal orientational metaphor. However, it is related to the position of central-peripheral. In the image, the male protagonist is depicted in the right bottom corner, while the two female protagonists are situated in the central position. Moreover, the male protagonist is portrayed as turning his head toward the two female protagonists, who are portrayed as talking to each other and walking in an opposite direction. The polar position metaphorically represents the emotion: Being Sad is Being Physically Isolated.

### 5.3. Sadness is down

**FIGURE 16:**
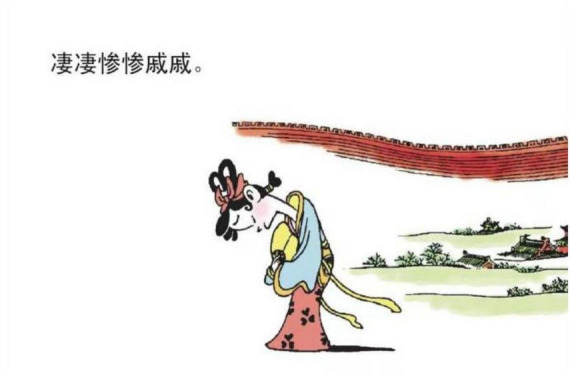
The third panel on Cai (2014, p. 30). Images reproduced with permission from Shan Dong People Press.

“SADNESS IS DOWN” is typically a type of orientational metaphor. Such metaphors are related to spatial orientation, which “organizes a whole system of concepts with respect to one another” (Lakoff and Johnson, 1980, p. 11). SADNESS IS DOWN is a spatial orientation metaphor of up-down, through the physical basis “dropping posture typically goes along with sadness and depression” (Lakoff and Johnson, 1980, p. 11). The physical basis for the metaphorical structure is also coherent with the value of stressing verticality in the culture. That is, in the Chinese cultural experience, being sad is considered a negative emotion, and being negative is oriented toward the direction of DOWN. Figure 16 is an example showing how the source domain is depicted in the comic book. It metonymically describes the posture of a female protagonist, who is situated in the borderland, dropping her posture, frowning, and closing her eyes. The dropping posture represents her feeling of sadness. Such posture appears many times in the comic book.

### 5.4. Other types of metaphors

In addition to the major metaphors discussed, there are also SADNESS IS DARK, SADNESS IS BITTERSWEET DRINK, SADNESS IS BEING CONFRONTED WITH AN OPPONENT, and SADNESS IS LACK OF HEAT. Those types appear less frequently, among which SADNESS IS BITTERSWEET DRINK, which is worth an exploration. In many images (e.g., Panel 1 in Figure 1), the drink is rice wine, which is metonymically presented by a small cup. In the Chinese context, a single cup of rice wine is always considered bitter. Such experience is perceived as projecting the emotion of sadness.

In accordance with the discussion, we could understand that during the metaphorical structure, some specific features of the source domains are extracted. It is based on those features that we can identify the aspect of the emotion of sadness. As Lakoff and Johnson (1980, p. 217) state, metaphor is “typically based on cross-domain correlations in our experience, which give rise to the perceived similarities between the two domains within the metaphor.” Moreover, the metaphorical mapping of the source domain is in fact a selection of properties from a level of categories that matches the target domain. It reflects the laws of human cognition. The metaphorical depiction of an aspect of an object foregrounds the feature of sadness and thus enhances the understanding of that aspect.

## 6. Chinese cultural characteristics of the conceptual metaphor of sadness

Sadness is viewed as one of the primary emotions, which is hard-wired in the human neuroanatomy (Turner, 2000). As has been stressed by Lakoff and Johnson (1980), the perceived similarities between the source and the target domains are not only based on physical factors but also on cultural factors. Such cultural factors would provoke culture-specific metaphor structures, which have been demonstrated in the present study. This section provides an elaboration on the cultural characteristics of the emotion metaphor in the traditional Chinese context.

The analysis finds that the source domains that are used to bring the emotion of sadness into reality are mostly liquid (river, sea, rain, etc.), birds, plants, and some natural and material substances. The findings foreground the Chinese philosophical systems of “Taoism” and “Buddhism,” two major streams of religions prevailing in the Song Dynasty. They are reflected in three specific aspects of the metaphor mechanism in the Chinese cultural context.

First, Taoism proposes the theory of YIN and YANG philosophy. YIN and YANG are generalizations of the attributes of the opposing sides of certain interrelated things or phenomena in nature. YANG is always used to describe the concepts of warmth, dryness, brightness, etc., and YIN is used to describe the concepts of coldness, wetness, darkness, etc. According to the theory, liquid and moon belong to the category of YIN. The features of such source domains used to represent the emotion of sadness reflect the philosophical thought of YIN and YANG is highly valued.

Second, Taoism advocates the philosophy of “the unity of humans and the universe” (Zhong et al., 2021). Based on the basic concept, human beings have come to understand that the universe is composed of human beings and everything in the natural world. As Tzu (2004) Tao Te Ching states, these are the four greatnesses of the Kosmos, and of them, Man is one. Man patterns after Earth, Earth patterns after Heaven, Heaven patterns after the Tao, and the Tao patterns after that which is natural. Human beings realize the inseparable relationship with nature. The essence of human beings and nature is in harmony with each other. Their emotions are thus always expressed through employing the features of natural resources. This is the reason why the birds, fading flowers, and falling leaves are exploited as the source domains. With the intrinsic associations with human beings in nature, the emotion of sadness is perceived.

Third, “kindness”[Skt. Maitrī] and “compassion”[Skt. Karuṇā] are two fundamental doctrines in Buddhism, while Buddhism considers death and departure with the loved ones as two recognized sources of compassion revealed in the “truth of suffering” (Lock and Linebarger, 2018, p. 24). As *Song Ci* unfolds the story leading to an unhappy ending, traces of leaving, places of burying ancestors or large empty space representing distance can be adopted as a concrete image to map the emotion of sadness (e.g., Figures 3–5, 13, 14, 16). These metaphoric devices are usually presented together in panels but the first one, forming a metaphor mix that seems to comply with the sequential flow of comic narratives tends to reach its peak after the context is established (Cohn, 2019).

In short, the conceptual metaphor system comes from living experience. It cannot be formed spontaneously in isolation from the context. Context is what the anthropologist Malinowski calls the cultural context, which includes the universal experiences shared by human beings, and the cultural values specific to people and communities. Different cultural contexts shape their values toward certain phenomena and things and their nature. Metaphors thus reflect the cultural patterns of the physical world we inhabit.

## 7. Conclusion

Conceptual metaphor theory is a widely applicable theory in various linguistic forms, both verbal and visual, that has drawn burgeoning scholarly interest in the past decades, with metaphors of emotion constantly being placed under the spotlight for its natural connection with physical experience. However, the representation of the emotion of sadness in visual discourse has received insufficient attention. By following established procedures of conceptual metaphor identification, we have found two dominant visual metaphors that bear evident Chinese cultural characteristics on top of five universal ones. Quantitatively speaking, BEING SAD IS BEING CONFRONTED WITH NATURAL FORCE, and BEING SAD IS BEING PHYSICALLY ISOLATED amount to be the two most prevalent source domains with frequencies of 48 and 44 in our compiled dataset. Qualitatively speaking, the source metaphors identified are discussed in connection to the theoretical basis in CMT and experience universal in the human world or unique in the Chinese cultural context. In addition, we further extend our discussion to the underlying Chinese cultural notions and philosophies behind the Chinese conceptual metaphor of sadness.

There are, nevertheless, some limitations of our study that should be noted. First, a wider range of investigation into the adaptive works of ancient Chinese classics is necessary to reach a more widely applicable conclusion about how metaphors of sadness are mapped visually. In addition, the interpretation of images, though we have made an effort to standardize the coding process, can be somewhat subjective and vary according to personal understanding and experience.

## Data availability statement

The raw data supporting the conclusions of this article will be made available by the authors, without undue reservation.

## Author contributions

SW: Conceptualization, Data curation, Formal Analysis, Investigation, Writing – original draft, Writing – review and editing. ZZ: Data curation, Formal Analysis, Investigation, Methodology, Software, Visualization, Writing – original draft, Writing – review and editing. SC: Methodology, Supervision, Writing – review and editing.

## Footnotes

1
http://www.wagsoft.com/ImageTool/download.html
